# Seroprevalence and Risk Factors of Bluetongue Virus Infection in Tibetan Sheep and Yaks in Tibetan Plateau, China

**DOI:** 10.1155/2017/5139703

**Published:** 2017-04-23

**Authors:** Jian-Gang Ma, Xiao-Xuan Zhang, Wen-Bin Zheng, Ying-Tian Xu, Xing-Quan Zhu, Gui-Xue Hu, Dong-Hui Zhou

**Affiliations:** ^1^State Key Laboratory of Veterinary Etiological Biology, Key Laboratory of Veterinary Parasitology of Gansu Province, Lanzhou Veterinary Research Institute, Chinese Academy of Agricultural Sciences, Lanzhou, Gansu 730046, China; ^2^College of Animal Science and Technology, Jilin Agricultural University, Changchun, Jilin 130118, China; ^3^College of Agriculture, Yanbian University, Yanji, Jilin 133002, China; ^4^Jiangsu Co-Innovation Center for the Prevention and Control of Important Animal Infectious Diseases and Zoonoses, Yangzhou University College of Veterinary Medicine, Yangzhou, Jiangsu 225009, China

## Abstract

Bluetongue (BT), caused by bluetongue virus (BTV), is an arthropod-borne viral disease in ruminants. However, information about BTV infection in yaks in China is limited. Moreover, no such data concerning BTV in Tibetan sheep is available. Therefore, 3771 serum samples were collected from 2187 Tibetan sheep and 1584 yaks between April 2013 and March 2014 from Tibetan Plateau, western China, and tested for BTV antibodies using a commercially available ELISA kit. The overall seroprevalence of BTV was 17.34% (654/3771), with 20.3% (443/2187) in Tibetan sheep and 13.3% (211/1584) in yaks. In the Tibetan sheep group, the seroprevalence of BTV in Luqu, Maqu, Tianzhu, and Nyingchi Prefecture was 20.3%, 20.8%, 20.5%, and 19.1%, respectively. The seroprevalence of BTV in different season groups varied from 16.5% to 23.4%. In the yak group, BTV seroprevalence was 12.6%, 15.5%, and 11.0% in Tianzhu, Maqu, and Luqu counties, respectively. The seroprevalence in different seasons was 12.6%, 15.5%, 15.4%, and 9.0% in spring, summer, autumn, and winter, respectively. The season was the major risk factor concerning BTV infection in yaks (*P* < 0.05). The date of the BTV seroprevalence in Tibetan sheep and yaks provides baseline information for controlling BT in ruminants in western China.

## 1. Introduction

Bluetongue virus (BTV), a member of the genus* Orbivirus*, family Reoviridae, is the causative agent of bluetongue (BT), an infectious, noncontagious, arthropod-borne viral disease, which can infect a wide range of wild and domestic ruminants [1]. The first case of BT in sheep in India was reported in 1964 [1]. This pathogen was firstly recorded in sheep in China in 1979 [2]. Because BTV can cause a severe hemorrhagic disease with high morbidity, it is listed as a notifiable disease by Office International des Epizootics (OIE) [1]. Transmission of BTV is mainly through biting of blood-feeding insect vectors of the genus* Culicoides* (Diptera: Ceratopogonidae). BTV infection in sheep and wild ruminants usually presents as symptoms of fever, nasal discharge, drooling of saliva, oral lesion, facial edema, depression, anorexia, and muscle weakness. In contrast, goats and cattle may be asymptomatic [3].

Recently, a large number of BTV surveys have been conducted worldwide. In China, research focused on sheep, goats, and cattle [4, 5]. According to the literature published in a Chinese journal, the abortion rates of yaks were 21.39% based on an investigation of 104 farms in Qinghai Province, which could be caused by BTV and other pathogens [6]. However, no such data concerning BTV in Tibetan sheep are available, and only one case of BTV infection in yak was reported in Qinghai Province [7]. Tibetan sheep* (Ovis aries)* and black and white yaks* (Bos grunniens)* are important semiwild animals in China, and they mainly live in Tibetan Plateau which has low air pressure, lower temperature, and oxygen content. The white yaks (~49,400) is a unique yak breed living only in Tianzhu Tibetan Autonomous County, Gansu Province, northwestern China. More importantly, Tibetan sheep and black and white yaks have become the most important income source for local Tibetans. Therefore, seroprevalence of BTV infection in Tibetan sheep and black and white yaks in Tibetan Plateau, China, was conducted in this study.

## 2. Materials and Methods

### 2.1. Study Area

**Figure 1:**
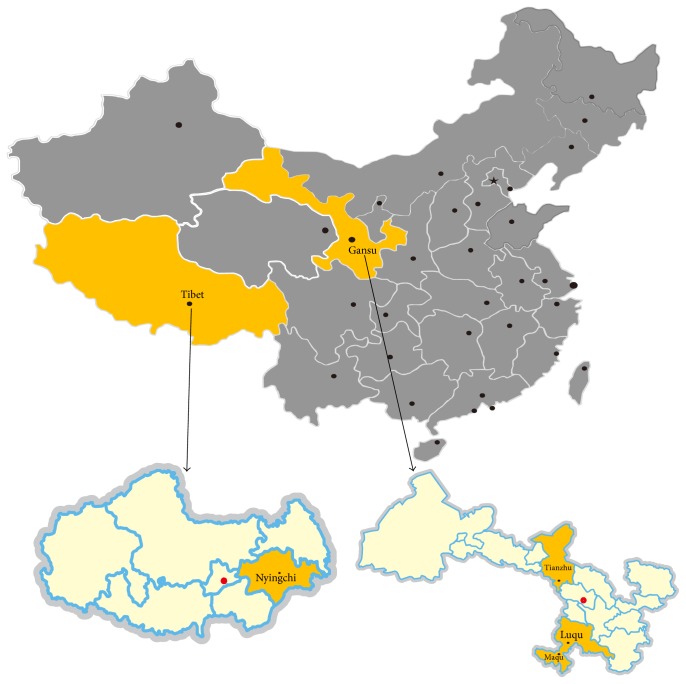
A map of China showing the geographical regions in Tibet and Gansu Provinces where farmed Tibetan sheep and yaks were sampled.

The study was conducted in two provinces in western China, namely, Gansu (32°31′′–42°57′′N, 92°13′′–108°46′′E) and Tibet (26°50′′–36°53′′N, 78°25′′–99°06′′E), the Tibetan Plateau, with an average elevation of 4000 metres (Figure 1). These regions have plateau continental climate, with short summer and long winter, and the average annual temperature is only 0°C.

### 2.2. Sample Collection

This study was approved by the Animal Ethics Committee of Lanzhou Veterinary Research Institute, Chinese Academy of Agricultural Sciences. A total of 3771 blood samples from 2187 Tibetan sheep (962 from Tianzhu with the elevation above 2,000 metres, 182 from Luqu with an average elevation of 3,500 metres, 588 from Maqu with an average elevation of 3,700 metres, and 455 from Nyingchi with an average elevation of 3,000 metres) and 1584 yaks (974 from Tianzhu, 146 from Luqu, and 464 from Maqu) were randomly collected between April 2013 and March 2014. All the blood samples were transported directly to the laboratory in Lanzhou Veterinary Research Institute, Chinese Academy of Agricultural Sciences, Lanzhou, Gansu Province, China. Serum was obtained through centrifugation at 1000*g* for 5 min. The serum was separated and stored at −20°C until analysis. Information about breed, geographic origin, gender, age, and season was obtained from local farmers and is listed in Table 1.

### 2.3. Serological Assay

Serum samples were examined using a commercially available c-ELISA kit (Veterinary Medical Research and Development (VMRD) Inc., Pullman, Washington, USA) to screen for BTV-specific IgG antibodies following the manufacturer's instructions [8]. The samples were considered positive when the optical density is less than or equal to 50% of the mean of the negative controls. Then the serum samples with positive or doubtful results were retested.

### 2.4. Data Analysis

The variation in seroprevalence of BTV-infected Tibetan Sheep and Yaks of different variables including breed, geographic origin, gender, age, and season was analyzed by *χ*^2^ test using SAS version 9.1 (SAS Institute Inc., USA). *P* value < 0.05 was considered as statistically significant. Odds ratios (ORs) and their 95% confidence intervals (95% CIs) were also calculated.

## 3. Results

Six hundred and fifty-four (17.3%) out of 3771 serum samples were seropositive for BTV infection using an indirect ELISA test. The seroprevalence ranged from a minimum of 12.6% among the white yaks to a maximum of 20.3% among the Tibetan sheep. In the Tibetan sheep group, the seroprevalence of BTV in Luqu, Maqu, Tianzhu, and Nyingchi was 20.3%, 20.8%, 20.5%, and 19.1%, respectively, and the detailed information about each group is shown in Table 1. In the yak group, BTV seroprevalence was 12.6%, 15.5%, and 11.0% in Tianzhu, Maqu, and Luqu counties, respectively, and the detailed information about each group is shown in Table 1.

According to conditional forward stepwise logistic regression, a significant difference was found between Tibetan sheep and yak groups (*P* < 0.05), for which the OR was 1.653 (95% CI 1.383–1.976) (Table 2). For the Tibetan sheep group, logistic regression analysis has shown that the season, gender, age, and region were not the significant risk factors (*P* > 0.05). For the yak group, logistic regression analysis showed that only season was significantly associated with BTV infection (*P* < 0.05) (Table 2).

## 4. Discussion

In the present study, the overall BTV seroprevalence in the examined Tibetan sheep and yaks was 17.3%. This rate is higher than the 3.53% reported in yaks in Qinghai Province, China [7], and 9.3% among the domestic ruminants in Northern Kerala, India [3]. However, it is lower than the 27.9% prevalence reported in small ruminants in Nepal [2], 33.13% in sheep and goat in South Bengal [9], 43.68% in ruminants in Jharkhand, India [10], 45.20% among domestic ruminants in the highlands of Nepal [11], and 96.7% in buffaloes and cattle in selected provinces in Lao People's Democratic Republic [12]. Many factors, including the diagnostic methods, climatic conditions, geographical conditions, species/breeds, sample sizes, and sanitation, may contribute to such differences.

In the present survey, Tibetan sheep showed a higher BTV seroprevalence than yaks, and the difference was statistically significant (*P* < 0.05). This result is consistent with a previous study which demonstrated that sheep are more susceptible to BTV [3]. It is well known that* Culicoides* midges are the most important transmitting vector for BTV. Moreover, the* Culicoides* midges are seasonal; they began to be active in spring and are most active in summer in these regions [13]. This seasonal exposure fits with the seroprevalence data in yaks in this study. Hence, the seasons were undoubtedly the risk factor for BTV infection in yaks.

The present study has shown that seasons are highly related to BTV infection in yaks (*P* < 0.05). Yaks had a 1.87 times higher risk for infection with BTV in summer compared to winter (OR = 1.87, 95% CI = 1.17–3.00) and a 1.85 times higher risk for infection with BTV in autumn compared to winter (OR = 1.85, 95% CI = 1.18–2.91), but it was not different significantly between spring and winter using multivariable analysis (Table 2). Such difference may be due to the fact that the Tibetan sheep and yaks were slaughtered during September and November every year, and the unhealthy and adult animals would be slaughtered first. Hence, the seroprevalence was lower in winter than in other seasons. However, the* Culicoides* midges would breed when the winter is passed, and they could spread the virus again. Although the season was not considered as the risk factor for Tibetan sheep, the seroprevalence in summer was higher than other seasons. These findings suggest that seasons should be considered when carrying out control programs in the investigation areas.

In the present investigation, no significant difference in BTV seroprevalence was observed among Tibetan sheep and yaks of different ages. This might be because these animals stayed in the same location.

In summary, the present study revealed that BTV infection is widespread in Tibetan sheep (20.3%) and yaks (13.3%) in Gansu and Tibet, western China. This is also the first report of BTV seroprevalence and risk factors in Tibetan sheep in China. The logistic regression analysis showed that the species was the risk factor concerning BTV infection. Season is considered as the risk factor of BTV infection in yaks. Hence, we should pay more attention to controlling* Culicoides* midges in warm seasons, especially in summer and autumn. These data provide baseline information for the control of BTV infection in Tibetan sheep and yaks.

## Figures and Tables

**Table 1:** Seroprevalence and risk factors of bluetongue virus (BTV) infection in Tibetan sheep and yaks in Tibetan Plateau, western China, by enzyme-linked immunosorbent assay (ELISA).

Variable	Tibetan sheep				Yak			
	Number	Number	%	*P* value	Number	Number	%	*P* value
	Tested	Positive			Tested	Positive		
Age group (years)								
1 or less	447	82	18.3	0.6418	286	38	13.3	0.1148
1-2	413	90	21.8		292	38	13.0	
2–4	1116	229	20.5		521	83	15.9	
>4	211	42	19.9		485	52	10.7	
Gender								
Male	638	140	21.9	0.2076	471	70	14.9	0.2402
Female	1549	303	19.6		1113	141	12.7	
Region								
Tianzhu	962	197	20.5	0.9230	974	123	12.6	0.2178
Luqu	182	37	20.3		146	16	11.0	
Maqu	588	122	20.8		464	72	15.5	
Nyingchi	455	87	19.1					
Season								
Spring	480	103	21.5	0.1708	428	54	12.6	0.0293
Summer	398	93	23.4		354	55	15.5	
Autumn	479	98	20.5		467	72	15.4	
Winter	375	62	16.5		335	30	9.0	
No information	455	87	19.1		—	—	—	
Breed								
White yak	—	—	—	—	974	123	12.6	0.3055
Black yak	—	—	—	—	610	88	14.4	
Total	2187	443	20.3	—	1584	211	13.3	—

**Table 2:** Odds ratios of the risk factors for bluetongue virus (BTV) seroprevalence in Tibetan sheep and yaks (*n* = 3771).

Factor	Group	Prevalence (%)	OR	95% CI	*P* value	
					Univariable analysis	Multivariable analysis
Breed	Yak	13.3	Reference			Reference
	Tibetan sheep	20.3	1.653	1.383–1.976	<0.0001	<0.0001
Season (in yaks)	Winter	9.0	Reference			Reference
	Spring	12.6	1.468	0.916–2.352	0.0293	0.1088
	Summer	15.5	1.870	1.166–3.000		0.0086
	Autumn	15.4	1.853	1.180–2.910		0.0067
